# Drug discovery for male subfertility using high-throughput screening: a new approach to an unsolved problem

**DOI:** 10.1093/humrep/dex055

**Published:** 2017-03-16

**Authors:** Sarah J. Martins da Silva, Sean G. Brown, Keith Sutton, Louise V. King, Halil Ruso, David W. Gray, Paul G. Wyatt, Mark C. Kelly, Christopher L.R. Barratt, Anthony G. Hope

**Affiliations:** 1 Reproductive and Developmental Biology, University of Dundee, Ninewells Hospital, Dundee DD1 9SY, UK; 2 Assisted Conception Unit, Ninewells Hospital, Dundee DD1 9SY, UK; 3 School of Science Engineering and Technology, University of Abertay, Dundee DD1 1HG, UK; 4 Drug Discovery Unit, School of Life Sciences, University of Dundee, Dundee DD1 5EH, UK

**Keywords:** sperm function, male infertility, calcium, drug discovery, high-throughput screening

## Abstract

**STUDY QUESTION:**

Can pharma drug discovery approaches be utilized to transform investigation into novel therapeutics for male infertility?

**SUMMARY ANSWER:**

High-throughput screening (HTS) is a viable approach to much-needed drug discovery for male factor infertility.

**WHAT IS KNOWN ALREADY:**

There is both huge demand and a genuine clinical need for new treatment options for infertile men. However, the time, effort and resources required for drug discovery are currently exorbitant, due to the unique challenges of the cellular, physical and functional properties of human spermatozoa and a lack of appropriate assay platform.

**STUDY DESIGN, SIZE, DURATION:**

Spermatozoa were obtained from healthy volunteer research donors and subfertile patients undergoing IVF/ICSI at a hospital-assisted reproductive techniques clinic between January 2012 and November 2016.

**PARTICIPANTS/MATERIALS, SETTING, METHODS:**

A HTS assay was developed and validated using intracellular calcium ([Ca^2+^]_i_) as a surrogate for motility in human spermatozoa. Calcium fluorescence was detected using a Flexstation microplate reader (384-well platform) and compared with responses evoked by progesterone, a compound known to modify a number of biologically relevant behaviours in human spermatozoa. Hit compounds identified following single point drug screen (10 μM) of an ion channel-focussed library assembled by the University of Dundee Drug Discovery Unit were rescreened to ensure potency using standard 10 point half-logarithm concentration curves, and tested for purity and integrity using liquid chromatography and mass spectrometry. Hit compounds were grouped by structure activity relationships and five representative compounds then further investigated for direct effects on spermatozoa, using computer-assisted sperm assessment, sperm penetration assay and whole-cell patch clamping.

**MAIN RESULTS AND THE ROLE OF CHANCE:**

Of the 3242 ion channel library ligands screened, 384 compounds (11.8%) elicited a statistically significant increase in calcium fluorescence, with greater than 3× median absolute deviation above the baseline. Seventy-four compounds eliciting ≥50% increase in fluorescence in the primary screen were rescreened and evaluated further, resulting in 48 hit compounds that produced a concentration-dependent increase in [Ca^2+^]_i_. Sperm penetration studies confirmed *in vitro* exposure to two hit compounds (A and B) resulted in significant improvement in functional motility in spermatozoa from healthy volunteer donors (A: 1 cm penetration index 2.54, 2 cm penetration index 2.49; *P* < 0.005 and B: 1 cm penetration index 2.1, 2 cm penetration index 2.6; *P* < 0.005), but crucially, also in patient samples from those undergoing fertility treatment (A: 1 cm penetration index 2.4; *P* = 0.009, 2 cm penetration index 3.6; *P* = 0.02 and B: 1 cm penetration index 2.2; *P* = 0.0004, 2 cm penetration index 3.6; *P* = 0.002). This was primarily as a result of direct or indirect CatSper channel action, supported by evidence from electrophysiology studies of individual sperm.

**LIMITATIONS, REASONS FOR CAUTION:**

Increase and fluxes in [Ca^2+^]_i_ are fundamental to the regulation of sperm motility and function, including acrosome reaction. The use of calcium signalling as a surrogate for sperm motility is acknowledged as a potential limitation in this study.

**WIDER IMPLICATIONS OF THE FINDINGS:**

We conclude that HTS can robustly, efficiently, identify novel compounds that increase [Ca^2+^]_i_ in human spermatozoa and functionally modify motility, and propose its use as a cornerstone to build and transform much-needed drug discovery for male infertility.

**STUDY FUNDING/COMPETING INTEREST(S):**

The majority of the data were obtained using funding from TENOVUS Scotland and Chief Scientist Office NRS Fellowship. Additional funding was provided by NHS Tayside, MRC project grants (MR/K013343/1, MR/012492/1) and University of Abertay. The authors declare that there is no conflict of interest.

**TRAIL REGISTRATION NUMBER:**

N/A.

## Introduction

Infertility is a significant global problem affecting ~1 in 7, or ~80 million couples (Slama *et al*., 2012; Datta *et al*., 2016). Although the causes of infertility are heterogeneous, male factor is now the leading cause and accounts for at least 50% of cases (HFEA, 2014). Asthenozoospermia (poor/dysfunctional sperm motility) is the commonest disorder in this population (Curi *et al*., 2003), yet there is no drug a man can take, nor that can be added to his sperm *in vitro*, to improve fertility (Barratt *et al*., 2011). Couples instead rely on Artificial Reproduction Technology (ART), such as IVF and ICSI, which is expensive, invasive and not without risk. Despite this, year-on-year, ART is increasingly utilized worldwide (Kupka *et al*., 2014, 2016; Sunderam *et al*., 2014). There is clearly an unmet need for the development of alternate treatment strategies for male factor infertility, and a demand for focussed treatment options to improve sperm motility.

Drug discovery is a lengthy, high-risk and expensive process, estimated to take at least 12 years and cost upwards of $2.6 billion for each drug to be successfully approved for clinical use (DiMasi *et al*., 2016). Computational chemistry and molecular mechanics are commonly utilized approaches in drug discovery, and use predictive modelling of molecular structure and properties to artificially design potential novel therapeutics. In the absence of a known specific molecular or receptor target, compound library high-throughput screening (HTS) is an alternative approach that has developed in recent decades (Miller, 2006). HTS provides an important source of hits for drug discovery programmes (Broach and Thorner, 1996), but its success clearly relies either on the diversity, or intended focus, of the library collection screened, as well as the diversity screened within the defined territory of the library itself, aiming to maximize coverage of ‘lead-like chemical space’. However, a fundamental hurdle to development of new drugs, either for male fertility or contraceptive use, is the lack of appropriate assay systems to allow rapid screening of large and diverse pharma libraries (Carlson *et al*., 2009). The challenge of the unique size, shape and motility of spermatozoa has instead resulted in current strategies based on direct assessment of sperm motility and function, which is not feasible in the context of a drug discovery programme due to the prohibitive costs of time and resources (Tardif *et al*., 2014).

Sperm do not transcribe nor translate and instead rely on post-translational modification of proteins as primary signalling mechanisms (Grootegoed *et al*., 2000). For example, cyclic AMP-dependent phosphorylation of flagellar proteins is required for initiation and maintenance of sperm motility (Tash and Means, 1982, 1983; San Agustin and Witman, 1994; Visconti *et al*., 1997). Ion channels and ionic gradients play key roles in orchestrating intracellular signalling pathways (Darszon *et al*., 2011). Increase and fluxes in intracellular calcium [Ca^2+^]_i_ are fundamental to the regulation of sperm motility and function, including changes in direction, hyperactivation and chemotaxis (Publicover *et al*., 2007; Darszon *et al*., 2011) and there is a plethora of evidence that calcium mobilization in sperm is related to fertilization *in vivo* and *in vitro* (Baldi *et al*., 2009; Publicover and Barratt, 2011; Alasmari *et al*., 2013a,b).

[Ca^2+^]_i_ is regulated by at least two processes in spermatozoa: mobilization of Ca^2+^ stored in the acrosome and neck region (Ho and Suarez, 2001, 2003; Ren *et al*., 2001; Ho *et al*., 2002; Quill *et al*., 2003; Marquez *et al*., 2007) and/or transmembrane flux through Ca^2+^ permeable ion channels and transporters. In mice, the main source of Ca^2+^ entry appears to be *via* pH-dependent CatSper (Cation channel of Sperm) channels in the plasma membrane of the flagellar principal piece. CatSper is also essential for male fertility; sperm from CatSper knock-out mice are motile but cannot hyperactivate, rendering them unable to migrate to or within the oviduct (Carlson *et al*., 2003; Ho *et al*., 2009) and unable to fertilize oocytes, even by IVF (Ren *et al*., 2001). Further evidence that CatSper is critical to sperm function comes from the identification of natural mutations affecting CatSper gene family members (‘CatSper 1 and 2’) that are associated with oligoasthenoteratozoospermia (Hildebrand *et al*., 2010; Smith *et al*., 2013). Ca^2+^ entry into human sperm in response to progesterone (a product of cumulus cells) is *via* non-genomic activation of CatSper channels (Lishko *et al*., 2011; Strunker *et al*., 2011; Williams *et al*., 2015). CatSper activation by organic molecules that appear to evoke chemotaxis has also been described, and it is therefore proposed that this ion channel acts as a polymodal chemosensor that integrates multiple chemical cues from the female reproductive tract and directs sperm towards the egg (Brenker *et al*., 2012). Pharmacological agents, including direct or indirect CatSper agonists, could offer huge potential for development of new treatments for male subfertility.

**Figure 1:**
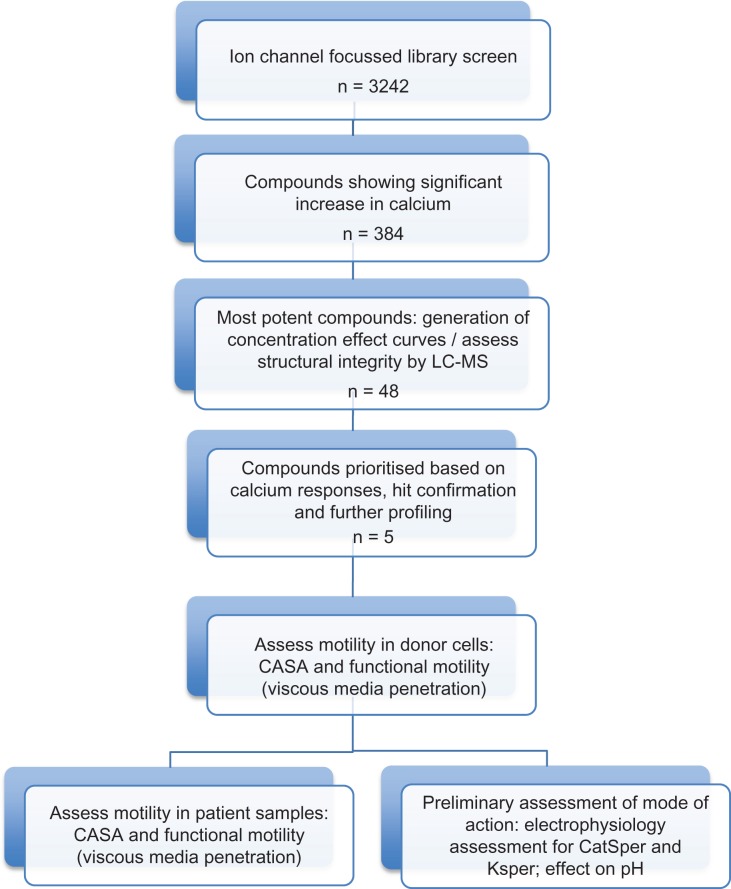
Schematic of research strategy. CASA, computer-assisted sperm analysis; LC–MS, liquid chromatography and mass spectrometry; CatSper, Cation channel of Sperm; KSper, K^+^ current that controls sperm membrane potential.

Given that calcium plays a fundamental role in sperm physiology, the aim of this study was to develop and validate a HTS assay using a calcium reporting system to allow screening of large libraries of drug discovery compounds to identify potential leads that may also have a positive functional effect on sperm motility. Following development of a HTS assay we screened 3242 compounds from an ion channel-focussed library. Potential leads of interest were subject to detailed analysis, which initially involved assessment of motility responses in donors. The compounds that showed a positive response were then tested on a selected group of patient samples using computer-assisted sperm assessment (CASA) and functional motility assays. Additionally, electrophysiology was performed to examine if the selected compounds activated CatSper. A schematic identifying this strategy is presented (Fig. 1).

## Materials and Methods

### Ethical approval

Healthy volunteer research donors with normal sperm concentration and motility according to WHO 2010 criteria (Cooper *et al*., 2010) were recruited in accordance with the Human Fertilisation and Embryology Authority (HFEA) Code of Practice (version 8) under local ethical approval (13/ES/0091) from East of Scotland Research Ethics Service (EoSRES) REC 1. Under the same ethical approval, subfertile patients undertaking treatment at Assisted Conception Unit (ACU), Ninewells Hospital, Dundee Scotland, consented to research use of samples surplus to clinical requirement.

### Semen samples

Semen samples were collected by masturbation into a sterile plastic container after 2–5 days of sexual abstinence. Samples were used for analysis after liquefaction for ~30 min at 37°C, and processed within 1 h of production. Samples were obtained and analysed in line with suggested guidance for human semen studies (Bjorndahl *et al*., 2016).

### Sperm preparation

All chemicals were purchased from Sigma-Aldrich, UK. Cells were prepared by density gradient centrifugation (DGC), using non-capacitating medium (NCM; 1.8 mM CaCl_2_, 5.4 mM KCl, 0.8 MgSO_4_.7H_2_O, 116.4 mM NaCl, 1.0 mM Na_2_PO_4_.2H_2_O, 5.55 mM D-Glucose, 2.73 mM sodium pyruvate, 41.75 mM sodium lactate, 25 mM HEPES, 0.3% bovine serum albumin (BSA), pH 7.4)-buffered percoll. In all, ≤1.5 ml semen was added to the top layer of the density gradient (2 ml 40% underlaid with 2 ml 80%), and then centrifuged (300*g*, 20 min). The pellet was washed in NCM (500 g, 10 min). Cells were then resuspended in 5 ml bicarbonate-buffered medium to support capacitation (capacitation medium (CM); 3 mM CaCl_2_, 4.7 mM KCl, 1.0 mM MgSO_4_.7H_2_O, 106 mM NaCl, 1.5 mM Na_2_PO_4_.2H_2_O, 5.55 mM D-Glucose, 1.0 mM sodium pyruvate, 41.75 mM sodium lactate, 1.33 mM glycine, 0.68 mM glutamine, 0.07 mM taurine, 0.01 mM non-essential amino acids, 25 mM sodium bicarbonate, 0.3% BSA, pH 7.4–7.6) and incubated for 2–3 h (37°C, 5% CO_2_). Concentration and baseline motility assessment were performed using CASA before subjecting to experimental conditions.

Patient samples surplus to requirement for IVF treatment were also used for analysis. Commercially available media were used for sperm preparation by DGC (40%/80%), namely PureSperm^TM^ (Nidacom, Molndal, Sweden) diluted in Quinn's Advantage Medium with HEPES (SAGE In-Vitro Fertilisation, Pasadena, CA, USA). Following centrifugation (300*g*; 20 min), the pellet was washed by centrifugation at 500*g* for 10 min in 4 ml Quinn's Advantage Medium with HEPES. Supernatant was discarded and the pellet resuspended in Quinn's Advantage Fertilisation medium.

### Flexstation assay

Spermatozoa from two to four different donors were pooled together after sperm preparation in order to reliably obtain enough cells, and diluted to a density of 2.2 × 10^7^ cells/ml in Flexstation assay buffer (1× Hanks Buffered Salt Solution (HBSS, Invitrogen, Waltham, MA, USA), 20 mM HEPES, 0.5 mM probenecid, pH 7.4). Unless otherwise stated, an equal volume of the calcium-sensitive dye, Calcium 3 (Molecular Devices, Sunnyvale, CA, USA), made up to twice the manufacturer's recommended concentration, was added to the cells. Following incubation (37°C, 60 min), the sperm cells were recovered by centrifugation (700*g*, 5 min) and resuspended in Flexstation assay buffer at a density of 5 × 10^6^ cells/ml. Cells were plated on 384-well clear bottom, black wall assay plates (Greiner Bio One, UK) at a density of 2.5 × 10^5^ cells/50 μl/well. To ensure cells were restricted to the base of each well, the plates were centrifuged (700*g*, 5 min) at room temperature. Test compounds were prepared at a concentration of 50 μM on 384-well polypropylene plates (Greiner Bio One) as described below. The effect of compounds on spermatozoa [Ca^2+^]_i_ was measured using a Flexstation 3 (Molecular Devices). Baseline calcium-dependent fluorescence (excitation wavelength = 485 nm, emission wavelength = 525 nm, cut-off = 515 nm) was measured for 18 s. In all, 12.5 μl of each test compound was transferred to the assay plates using an internal 16-channel robotic pipette head and the resulting change in fluorescence monitored for a further 82 s.

### Compound screening

The University of Dundee Drug Discovery Unit (DDU) ion channel library comprises putative ion channel ligands assembled from ChEMBL database (Mok and Brenk, 2011). In all, 3242 compounds were prepared as stock solutions in dimethylsulphoxide (DMSO) at a concentration of 10 mM and supplied in 384-well Echo plates (Labcyte, Sunnyvale, CA, USA) for use in the screening campaign. In follow-up assays designed to determine the potency of hit compounds, the compounds were dispensed into 384-well polypropylene plates either from the compound library or from re-purchased material re-constituted in DMSO (10 mM), using an automated liquid handling workstation (JANUS, PerkinElmer, UK). A 3-fold serial dilution of each compound into DMSO and subsequent transfer into 384-well Echo plates was performed using the Biomek FX automated liquid handling workstation (Beckman Coulter, UK). Intermediate screening plates were generated by transferring 250 nl of compound into 384-well polypropylene plates using an Echo 550 accoustic dispenser (Labcyte) and diluting 200-fold in flexstation assay buffer (50 μl). Compound testing was performed on the Flexstation 3 as described above. All assay plates in the screen were subject to quality control analysis. The performance of the assay on each screening plate was also evaluated using internal controls, whereby the maximum and minimum calcium-mediated fluorescence was determined in the absence and presence of progesterone (10 μM), respectively.

### Motility assessment: CASA

Following preparation, spermatozoa were subjected to capacitating conditions for 2.5 h in a 5% CO_2_ humidified atmosphere. They were then mixed with either DMSO (vehicle; 1% final concentration) or compound (10 μM final concentration) and incubated further (37°C; 5% CO_2_ humidified atmosphere). Motility was evaluated using a CASA system (CEROS machine (version 12), Hamilton Thorne Research, Beverly, MA, USA) attached to an external microscope at set time intervals. Sperm kinematics were assessed under a negative phase contrast objective, using system parameter settings for analyses as previously described (Tardif *et al*., 2014). Spermatozoa were examined in four-chamber 20 μm deep slides (Vitrolife, Sweden), and 800–1000 sperm cells analysed per slide.

### Motility assessment: sperm penetration test

As previously described (Ivic *et al*., 2002), glass tubes (5 cm × 0.4 mm × 4 mm; Camlab Limited, Cambridge, UK) were loaded with methylcellulose (4000cp; Sigma-Aldrich M0512) by capillary action. The filled tubes were placed vertically into eppendorf tubes containing (i) 100 μl of spermatozoa (control), (ii) 100 μl spermatozoa and 1% DMSO (vehicle control), (iii) 100 μl spermatozoa and 3.6 μM progesterone (positive control) or (iv) 100 μl spermatozoa and 10 μM compound, and incubated at 37°C in 5% CO_2_ for 1 h. Number of spermatozoa was scored at 1 and 2 cm using an Olympus CX41 microscope (20× objective, final magnification ×200) (Olympus Corporation, Tokyo, Japan). Results were normalized to parallel untreated controls to allow comparison between different experiments, and expressed as a penetration index [number of spermatozoa observed with treatment/number of spermatozoa without treatment (control)].

### Patch-clamp electrophysiology

Electrophysiological responses of individual spermatozoa were investigated using whole-cell recording technique as previously described (Brenker *et al*., 2012; Mansell *et al*., 2014). Briefly, gigaohm seals were formed between the pipette and cytoplasmic droplet or midpiece of spermatozoa superfused with standard extracellular solution containing (mM): NaCl, 135; KCl, 5; CaCl_2_, 2; MgSO_4_, 1; HEPES, 20; Glucose, 5; Na pyruvate, 1; Lactic acid, 10; pH adjusted to 7.4 with NaOH which brought [Na^+^] to 154 mM. The transition to whole-cell configuration was achieved by light suction. CatSper-mediated monovalent currents (*I*_CatSper_) were recorded in sodium-based divalent-free extracellular solution (NaDVF) containing (mM): 140 NaCl, 40 HEPES, and one EGTA adjusted to pH 7.4 with NaOH and using Cs-based pipette solution containing (mM): Cs-methanesulphonate, 130; HEPES, 40; Tris–HCl, 1; EGTA, 3; EDTA, 2 mM, pH adjusted to 7.4 with CsOH. CatSper currents were evoked by successive 1 s depolarizing ramps from −80 to 80 mV from a holding potential of 0 mV.

K^+^ currents were recorded under quasi-physiological conditions (Mansell *et al*., 2014) using standard extracellular solution and standard pipette solution containing (mM): NaCl, 10; KCl, 18; K gluconate, 92; MgCl_2_, 0.5, CaCl_2_, 0.6; EGTA, 1; HEPES, 10; pH was adjusted to 7.4 using KOH which brought [K^+^] to 114 mM and [Ca^2+^] to 100 nM. K^+^ currents were evoked by successive 1 s depolarizing ramps from −92 to 68 mV (corrected for junction potential) at 1 Hz from a holding potential of −92 mV. All data are adjusted for cell capacitance, expressed as pA/pF.

### Cytoplasmic pH

Cytoplasmic pH was measured using 2′,7′-bis(2-carboxyethyl)-5,6-carboxyfluorescein (BCECF; ThermoFisher, Paisley, UK) and a FLUOstar omega reader (BMG Labtech, Offenburg, Germany). Donor sperm were prepared and capacitated as described above, and then incubated with 2 mM of the acetoxymethyl (AM)-coupled dye for 45 min. Free dye was removed and spermatozoa resuspended in assay buffer (BSA, Bicarbonate-free CM media buffered to pH 7.4 with HEPES). Fluorescence was monitored (excitation wavelength = 440/490 nm, emission wavelength = 530 nm) and quenched with manganese at the end of the assay period to allow subtraction of background fluorescence. NH_4_ or compound was added after 2 min. Extracellular calibration was used for cytoplasmic pH experiments; cells were lysed with 0.1% triton at the end of the assay period to record signal for pH 7.4. A fixed volume of 3.2% HCl was then added which reduced the pH to 6.7. As a comparator, intracellular calcium was measured using FURA2 (ThermoFisher) and FLUOstar omega reader as previously described (Williams *et al*., 2015). Progesterone-induced increments in the ratio of emission intensities (excitation wavelength = 340 nm, emission wavelength = 380 nm) were used to quantify changes in [Ca^2+^]_i_ concentration.

### Statistical analysis

Preliminary analysis of all HTS primary and potency raw data was performed using the AUC function within the SoftMax Pro analysis software (Molecular Devices) to quantitate agonist-evoked fluorescence. The criteria for data acceptance for each assay plate was based on robust statistics and defined as follows: (i) signal to Background ≥5 (AUC_max_/AUC_min_), (ii) Coefficient of Variance <12% (sMAD_max_/AUC_max_ × 100) and (iii) *Z*′ ≥ 0.5 (1−((3 × sMAD_max_) + (3 × sMAD_min_))/(AUC_max_ − AUC_min_)), where AUC_max_ was the median calcium-mediated fluorescence in the presence of progesterone (10 µM), AUC_min_ was the median fluorescence of all negative control signal, sMAD_max_ = 1.4826 (median absolute deviation (MAD) in the presence of progesterone) and sMAD_min_ = 1.4826 (MAD in the absence of progesterone).

Data were exported as a text file for further data processing and analysis in Activity Base version 7.3.1.4 (IDBS) and the percentage effect for each compound was normalized to the effect of progesterone (10 μM). All curve fitting was performed in Activity Base XE using the underlying ‘MATH IQ’ engine of XLfit version 5.1.0.0 from IDBS. The 4-parameter logistic dose–response curve *y* = *A* + (*B* – *A*)/1 + (10*^C^*/*x*)*^D^* was utilized for compound potency determination, where *A* is minimum response (AUC_min_), *B* is maximum response (AUC_max_), *C* is pEC_50_ and *D* is the Hill Slope. Potency was defined by reference to the negative log Molar value at the point of inflection of the sigmoidal concentration–response curve generated (pEC_50_). Database querying and report creation was undertaken using Reporter version 7.3.0 from IDBS.

Statistical comparisons for CASA kinematics and viscous penetration test (normalized against control) were made using unpaired *t*-tests. Unpaired *t*-tests were performed on currents obtained from whole-cell patch clamp. The statistical package Prism GraphPad (La Jolla, CA, USA) was used. *P* < 0.05 was considered significant.

## Results

### Flexstation assay development

**Figure 2:**
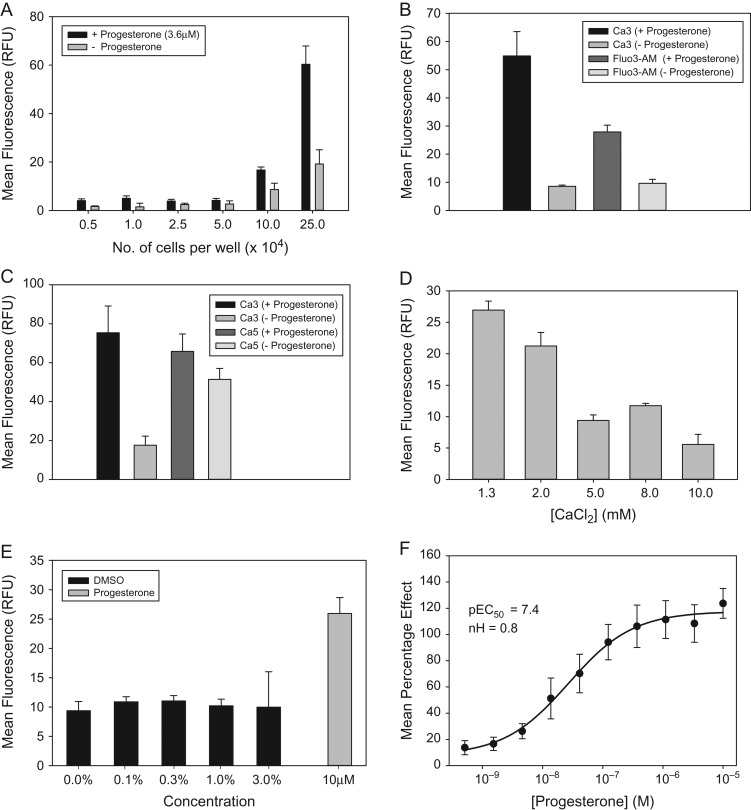
Flexstation assay development. Capacitated spermatozoa were pre-loaded with calcium-sensitive fluorescent dyes and progesterone evoked responses measured. Progesterone evoked fluorescence increased with increase in sperm cell concentration (**A**). Calcium 3 dye had a larger assay window compared with Fluo3-AM due to higher amplitude of fluorescence (**B**). Calcium 3 dye had a larger assay window compared with Calcium 5 dye, due to lower background fluorescence (**C**). Amplitude of the progesterone evoked response was inversely proportional to the extracellular calcium concentration (**D**). Progesterone elicited responses were unaffected by DMSO at concentrations up to 3% (v/v) (**E**). Progesterone evokes a concentration-dependent increase in intracellular calcium in human sperm cells (**F**).

A primary cell-based assay was optimized in 384-well plates, using a Flexstation 3 microplate reader to detect calcium fluorescence in motile human spermatozoa. The effect of progesterone (3.6 μM) on cytosolic calcium was assessed under a range of conditions. As expected, the calcium fluorescence elicited in response to progesterone increased with greater cell concentration (range 5000–250 000 cells in 50 μl per well; Fig. 2A). The amplitude of the response elicited by progesterone was also dependent upon the calcium-sensitive dye used (Fig. 2B and C), with the largest assay window being observed with Calcium 3. A smaller assay window was observed when cells were loaded with either Fluo3-AM, due to lower amplitude fluorescence response or Calcium 5, due to higher background fluorescence. All further experiments were therefore performed using Calcium 3. Fluorescence was measured at a range of extracellular calcium concentrations attempting to increase the amplitude of the evoked response, with a view to reducing cell number in the assay. However, an inverse relationship was observed between the progesterone response evoked and calcium concentration (Fig. 2D). Since all small molecules in the compound library are in solution in DMSO, we also confirmed that the assay was able to tolerate DMSO up to a concentration of 3% (v/v), which is in excess of the 0.1% (v/v) used in library screening (Fig. 2E). Conditions selected for subsequent compound testing were ~250 000 motile spermatozoa per well, suspended in 50 μl assay buffer containing 1.3 mM calcium chloride. Under these conditions, progesterone elicited a concentration-dependent increase in [Ca^2+^]_i_ in spermatozoa, pEC_50_ = 7.5 and Hill slope = 0.7 (Fig. 2F). These values are in agreement with those obtained by direct measurement of CatSper currents using electrophysiology (Strunker *et al*., 2011).

### Ion channel library screen

**Figure 3:**
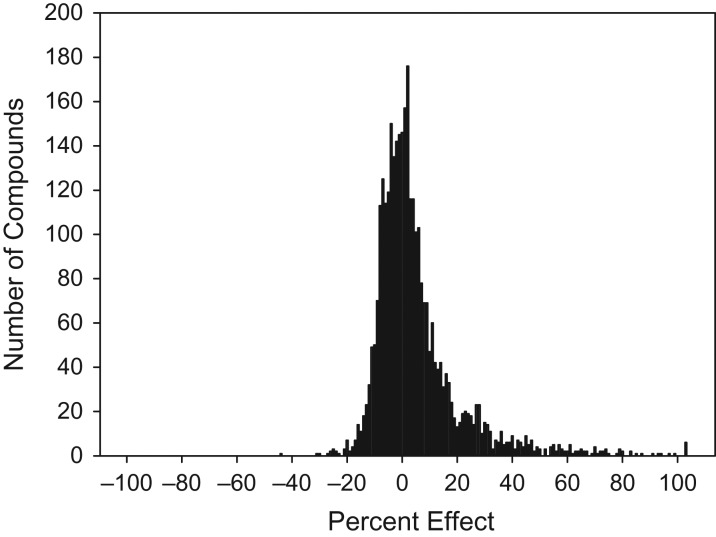
Ion channel library screen. Frequency histogram for 3242 compounds tested in the primary Flexstation screen. 384 compounds elicited a statistically significant increase in calcium fluorescence (>3 mean absolute deviation (MAD); 11.8%).

A focussed ion channel library screen was then performed using 80% fraction spermatozoa prepared by discontinuous DGC and subjected to capacitating conditions. Cells were pooled from multiple donors (*n* = 2–4 per plate). The primary assay proved to be highly reproducible (robust *Z*′ = 0.8 ± 0.1) and 3242 compounds were tested at a single concentration (10 μM). The primary screening data displayed a characteristic pseudo-normal distribution (Fig. 3) and 384 compounds (11.8%) elicited a statistically significant increase in calcium fluorescence, with greater than 3× MAD above the baseline. However, 74 of the most potent compounds were selected for the generation of concentration/effect curves based on a pragmatic cut-off of 50% increase in fluorescence. Of the 74 compounds re-tested, concentration–response curves were returned for 67, although structural integrity was only confirmed by liquid chromatography–mass spectrometry (LC–MS) for 48. Hit compounds were subsequently analysed and grouped by structure activity relationships (SARs). To facilitate further studies, concentration–response curves from hit compounds were visually inspected and the compounds prioritized based on their potential to elicit calcium response. The best agonists were coded A, B, C, D and E. These compounds were from several different chemical classes and structurally distinct from progesterone, and included a tertiary amide (A), piperidine amide (B), a secondary amide (C), thienopyrimidine (D) and quiniline (E). These five compounds were then used for hit confirmation and further profiling. Activity of each compound was confirmed in the flexstation assay with pEC_50_ values between 5.3 and 5.6 and Hill slopes of 0.9–2.5 (Table I; Supplementary Fig. S1).

**Table I:** Potency of five selected hit compounds from ion channel library screen.

Compound	Lab code	_*P*_EC_50_	Hill slope
DDD00104789	A	5.3	1.5
DDD00104960	B	5.4	1.0
DDD00105020	C	5.5	1.0
DDD00105498	D	5.6	1.3
DDD00106181	E	5.6	0.9

pEC50 and Hill slopes were determined from concentration effect curves generated for each compound (see also Supplementary Fig. S1).

### Motility assessment

**Figure 4:**
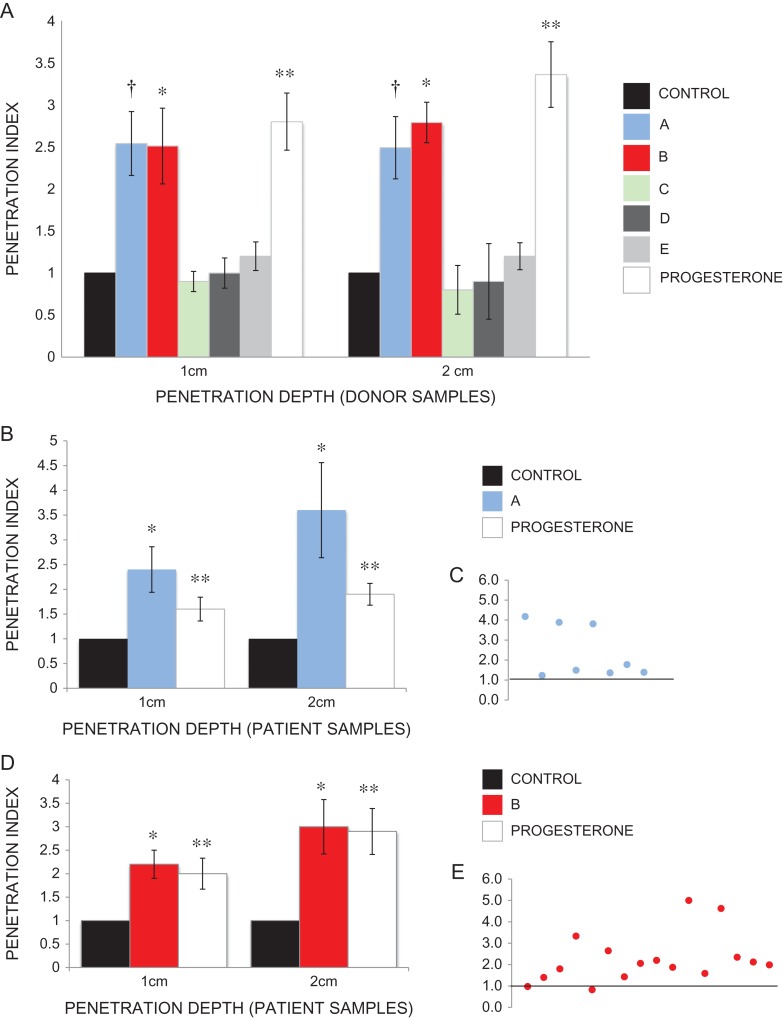
Sperm penetration assay. (**A**) Significant increase in functional motility seen in spermatozoa from healthy volunteer donors exposed to 10 μM compound A (1 cm penetration index 2.54, 2 cm penetration index 2.49; ^†^*P* < 0.005) and 10 μM compound B (1 cm penetration index 2.1, 2 cm penetration index 2.6; *P < 0.005). Other hit compounds elicited no increase in functional motility. 3.6 μM progesterone was used as a positive control (1 cm penetration index 2.80, 2 cm penetration index 3.36; **P < 0.005). (**B**) Significant increase in functional motility seen in spermatozoa from patient samples exposed to 10 μM compound A (1 cm penetration index 2.4; *P = 0.009, 2 cm penetration index 3.6; *P = 0.02). 3.6 μM progesterone was used as a positive control (1 cm penetration index 1.6; *P* = 0.03, 2 cm penetration index 1.9; **P = 0.001). (**C**) Individual patient responses to 10 μM compound A. Control (black line) = 1. (**D**) Significant increase in functional motility seen in spermatozoa from patient samples exposed to 10μM compound B (1 cm penetration index 2.2; *P = 0.0004, 2 cm penetration index 3.6; *P = 0.002). 3.6 μM progesterone was used as a positive control (1 cm penetration index 2.0; **P = 0.005, 2 cm penetration index 2.9; **P = 0.0005). (**E**) Individual patient response to 10μM compound B. Control (black line) = 1.

Initial motility assessment using CASA showed no significant improvement in total motility or progressive motility, nor individual kinematics, following addition of any compounds A–E to prepared spermatozoa from healthy volunteer donors (Supplementary Fig. S2). However, this was not unexpected, given the high level of motility characteristics in almost all prepared samples. Despite representing a key part of diagnostic semen analysis, it is widely accepted that motility assessment is an imperfect tool in predicting male fertility (Vasan, 2011). A sperm penetration assay was therefore used to assess and challenge functional motility of sperm, using 1% methylcellulose as viscous media (Kremer, 1965; Ivic *et al*., 2002). In all, 10 μM compound A significantly increased cell numbers penetrated into viscous media (1 cm penetration index 2.54, 2 cm penetration index 2.49; *P* < 0.005). In all, 10 μM compound B also significantly increased cell numbers penetrated into viscous media (1 cm penetration index 2.1, 2 cm penetration index 2.6; *P* < 0.005). There was no significant increase in penetration index with the other compounds tested (Fig. 4A).

Furthermore, a panel of patient samples (Supplementary Table S1) exposed to 10 μM compound A (*n* = 8) and 10 μM compound B (*n* = 17) also showed a significant increase in penetration index (A: 1 cm penetration index 2.4; *P* = 0.009, 2 cm penetration index 3.6; *P* = 0.02; Fig. 4B and C; B: 1 cm penetration index 2.2; *P* = 0.0004, 2 cm penetration index 3.6; *P* = 0.002; Fig. 4D and E). These samples were surplus to clinical requirements and donated for research on the day of IVF/ICSI, thus supporting a potential clinical application for this compound.

### Patch-clamp electrophysiology

**Figure 5:**
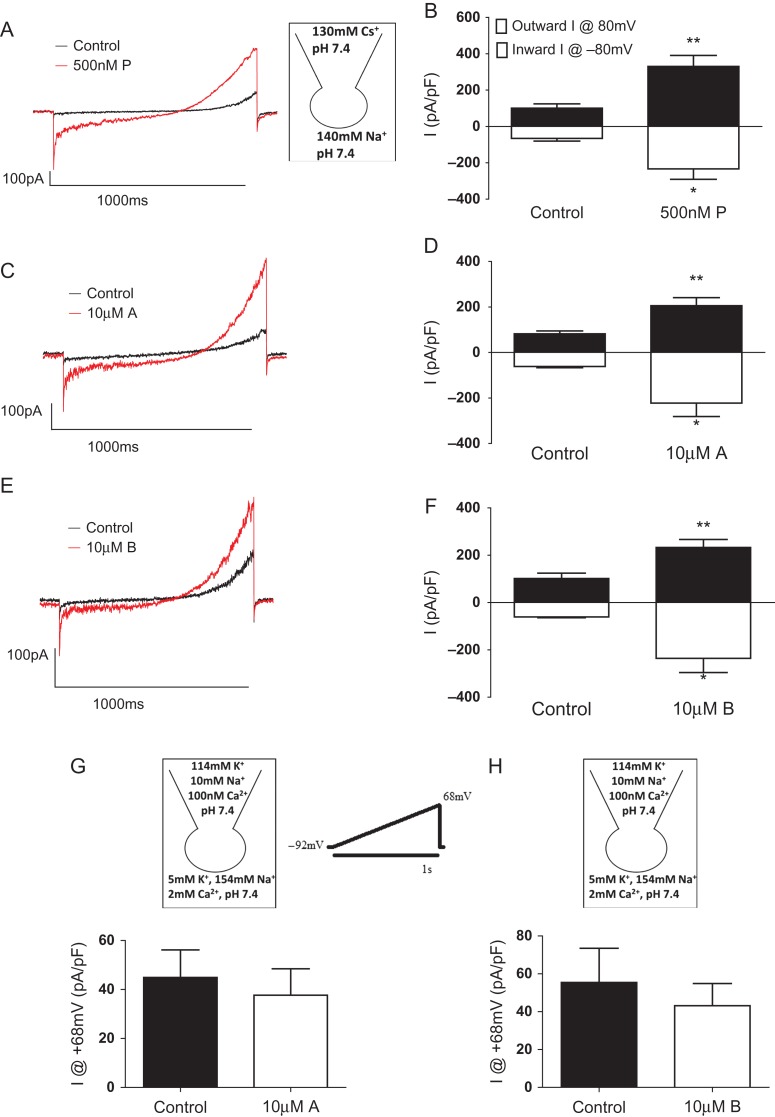
Patch-clamp electrophysiology. Compounds A and B selectively potentiate CatSper currents. Monovalent (NaDVF) CatSper currents (*I*_CatSper_) were evoked by ramp depolarization from −80 mV to 80 mV from a holding potential of 0 mV. (**A**) An example recording of 500 nM progesterone (P) potentiation of *I*_CatSper_. (**B**) Quantification of progesterone-induced potentiation of maximum inward and outward *I*_CatSper_ (*n* = 4). (**C**) An example recording of 10 μM compound A potentiation of *I*_CatSper_. (**D**) Quantification of A-induced potentiation of maximum inward and outward *I*_CatSper_ (*n* = 4). (**E**) An example recording of 10 μM compound B-induced potentiation of *I*_CatSper_. (**F**) Quantification of B-induced potentiation of maximum inward and outward *I*_CatSper_ (*n* = 5). K^+^ currents were recorded under quasi-physiological conditions and evoked by ramp depolarization from −92 to 68 mV from a holding potential of −92 mV (voltage protocol shown). Neither compound A (*n* = 4) nor B (*n* = 4) altered K^+^ current (measured at 68 mV, the Na^+^ reversal potential; **G, H**).

These two compounds (A and B) were then studied in a patch-clamp system, to assess individual human sperm responses (Mansell *et al*., 2014). Monovalent CatSper currents (*I*_CatSper_) were recorded under sodium-based divalent-free (NaDVF) conditions. As demonstrated previously (Lishko *et al*., 2011; Strunker *et al*., 2011), progesterone significantly potentiates *I*_CatSper_ (Fig. 5A and B). Similarly, these compounds significantly potentiated *I*_CatSper_ (Fig. 5C–F) with no effect on outward K^+^ currents (Brown *et al*., 2016) (Fig. 5G and H).

### Cytoplasmic pH

Given that alkalinization of cytoplasmic pH is a characterized activator for the CatSper complex, we used BCECF to track changes in intracellular pH following treatment with compounds A and B. However, no effect on pH was observed (Supplementary Fig. S3).

## Discussion

With the rationale that asthenozoospermia is the commonest problem underlying male subfertility (van der Steeg *et al*., 2011), sperm motility is an obvious target for development of new fertility treatments. However, motility screening is currently impossible to interrogate efficiently and accurately on a large scale, making this an unrealistic approach to drug discovery for male subfertility (Tardif *et al*., 2014). Despite the theoretical possibility of therapeutic manipulation to modify and improve sperm motility, effective treatment strategies for male factor infertility remain elusive. Nonetheless, the ability to treat motility defects in sperm could offer a viable alternative to IVF/ICSI for a significant cohort of patients, resulting not only in cost savings but reduced intensity of medical intervention and patient distress (van Rumste *et al*., 2014; Rooney and Domar, 2016). A key limitation in the quest for new treatments for male subfertility is the lack of detailed knowledge of the physiology and biochemical pathways underlying sperm motility and function, and a fundamental lack of obvious target for drug screening. However, specific loss of CatSper function is sufficient to compromise fertilizing capacity of human spermatozoa and is thought to represent an underlying factor in sperm dysfunction (Williams *et al*., 2015). Indeed, emerging data reveal the existence of multiple CatSper modulators, including synthetic endocrine disrupting chemicals (Tavares *et al*., 2013; Schiffer *et al*., 2014), which may negatively affect male fertility by effects on sperm motility and/or premature acrosome reaction.

This study shows that HTS can detect hit compounds with the potential to translate into identification of novel agents with positive effects on functional motility of sperm from subfertile patients. HTS directly examining motility effects, particularly when there is a need to rapidly screen thousands of compounds, is currently impracticable. Our screening approach instead focussed on a key aspect of sperm biology: modulation of [Ca^2+^]_i._ Having first established a HTS system, we then screened a 3242-compound ion channel-focussed library. Compounds that showed a positive response (demonstrating changes in calcium and motility) were subsequently tested on a selected group of patient samples using CASA and functional motility assays. Importantly, two compounds (A and B) showed a significant increase in functional motility in patient samples, thus demonstrating the potential of a HTS approach to discover novel compounds to enhance sperm function. Development of a HTS assay requires a balance between the necessity to screen a large number of compounds against the possibility and frequency of false positive/negative responses. Calcium was used in this screen primarily because it is known to be important for sperm motility, but also because it could be scaled up for HTS, as demonstrated by Schiffer and colleagues, who used a 384-microplate system to monitor calcium responses in human sperm to examine the action of 96 synthetic endocrine disrupting chemicals (Schiffer *et al*., 2014). However, whilst our data are very promising, calcium is only a surrogate of motility and has limitations. For example, some compounds generated an increase in calcium but no significant changes in functional motility. There are a number of reasons why this may be so. One explanation would be the nature of the calcium response examined in populations of spermatozoa over a short period of time. In this study (as in all studies of this type), cells are stimulated by a step change in agonist concentration. This induces an immediate [Ca^2+^]_i_ transient lasting ≈ 60 s which is followed by a [Ca^2+^]_i_ plateau. Effects on penetration of viscous medium will depend on effects exerted potentially over 10 s of minutes, whereas initial assessment of CASA characteristics is made 2–3 min after stimulation. Assay of the rapid [Ca^2+^]_i_ transient can provide an excellent HTS of CatSper activation but is likely to generate ‘false positives’ regarding functional motility—those instances when the Ca^2+^ transient is not followed by a significant plateau. Our previous work (Alasmari *et al*., 2013a,b and unpublished data) suggest that Ca^2+^ store mobilization downstream of CatSper activation may be important in determining the [Ca^2+^]_i_ plateau and consequent changes in functional motility, although the factors that determine whether this occurs are not yet clear.

CatSper channels appear to be the primary Ca^2+^ influx pathway in mammalian sperm (Brenker *et al*., 2012). By contrast, the contribution of conventional voltage-operated Ca^2+^ channels to Ca^2+^ influx is minimal (Zeng *et al*., 2013). Using patch-clamp electrophysiology and a pH-sensitive dye, we examined the mechanism by which two of the leading compounds (A and B) influence calcium. Both compounds A and B affected CatSper but there was no noticeable effect on KSper. As CatSper is partially regulated by pH we examined the effect on pH. There was minimal effect on intracellular pH, therefore compounds A and B are unlikely to activate CatSper *via* cellular alkalinisation. Both CatSper and KSper are unique to sperm, and have been identified as the primary ion channels in mice and humans by patch-clamp techniques (Lishko *et al*., 2011; Strunker *et al*., 2011; Mansell *et al*., 2014). Ion channels already represent a class of drug targets that have proven promising for numerous neurological and cardiovascular drug discovery applications (Bagal *et al*., 2013). However, as a class, ion channels remain underexploited in drug discovery, mainly due to poor selectivity or significant side effect profile or toxicities due to widespread tissue distribution of ion channels, coupled with the multitude of physiological consequences of their opening and closing. The existence of sperm-specific ion channels may avoid these issues, which makes an argument for this approach to drug discovery both valid and highly compelling.

What are the future practical uses of a HTS system for motile human spermatozoa? One potential use, as suggested previously, is to screen for compounds that can adversely affect sperm function, for example toxic chemicals in the environment (Schiffer *et al*., 2014), and/or to develop potential new targeted contraceptive approaches to deliberately negatively interfere with sperm function (Carlson *et al*., 2009). In these contexts, it may not be necessary to determine specific effects on sperm motility, as an adverse effect on calcium regulation could interfere with a plethora of other functional attributes of the human spermatozoon. With the notable lack of progress in male contraceptive research and identification of key targets (Amory, 2016) effective HTS also presents a powerful new approach. Our research strategy was to develop a tool to screen drug discovery library compounds to identify those that positively influence sperm function, specifically motility. Currently, we have only screened a small (3342 compounds) ion channel library but larger more comprehensive libraries can now be screened in a time and resource efficient way. Importantly, this will provide outputs that can then be used as starting points for a future hit optimization programme, and a more rigorous study of SARs, ultimately providing candidate drugs for clinical trials. Effective HTS is a cornerstone to drug discovery and an exciting step towards identification of new compounds that may be used to treat specific forms of sperm dysfunction. Further experiments examining relevant effects of these compounds are now necessary, for example, their effect on human IVF possibly using sibling oocytes as an experimental model. Critically, it is essential that experiments are done using human spermatozoa as the entry of calcium (and/or mobilization) into the mature human spermatozoon is very different in other mammals (Kaupp and Strunker, 2017) and the relevance of animal data to human cells is likely to be very limited.

In summary, we have developed an exciting first step towards utilization of HTS to drive forward drug discovery for male subfertility. This HTS screen has successfully examined an ion channel-focussed library, identifying compounds that increase [Ca^2+^]_i_ in motile human sperm cells from both healthy donors and, crucially, a small group of patients undertaking IVF/ICSI. These effects are likely modulated *via* CatSper, rather than pH, and positively impact on functional motility. Strategies aimed at further characterizing sperm ion transport and the control of specific functions that shape the unique and sophisticated behaviour of sperm offer exciting opportunities to increase our knowledge, as well as carrying clear translational possibilities to develop novel fertility treatments, and new hope to millions of infertile couples worldwide.

## Supplementary data

Supplementary data are available at *Human Reproduction* online.

## Authors’ roles

A.G.H., S.M.d.S., D.W.G., P.G.W. and C.L.R.B. were involved in the design of the study. S.M.d.S. obtained funding for the experiments, and was involved in the recruiting and consenting of patients and donors, preparation of media and donor samples and ion channel library compound high-throughput screening. H.R. and A.G.H. contributed to library compound screening. L.V.K., K.S. and M.K. undertook hit compound validation experiments. S.G.B. conducted the patch-clamp experiments and performed the detailed analysis of the electrophysiological data. The initial, interim and final manuscript was drafted by A.G.H., C.L.R.B. and S.M.d.S. All authors contributed to the construction, writing and editing of the manuscript. All authors approved the final manuscript.

## Funding

We acknowledge project grant funding from TENOVUS SCOTLAND in making this study possible (S.M.d.S.). Additional funding was provided by Chief Scientist Office/NHS research Scotland (S.M.d.S.), University of Abertay (sabbatical for S.G.B.), the Infertility Research Trust (C.L.R.B.) and Wellcome Trust (A.G.H., C.L.R.B.). Additional funding was provided by MRC project grants (MR/K013343/1, MR/012492/1; S.G.B., M.C.K., C.L.R.B.).

## Conflict of interest

C.L.R.B. is Editor in Chief of Molecular Human Reproduction and Chair of the World Health Organisation Expert Working Group on Diagnosis of Male infertility (2012–2016).

## Supplementary Material

Supplementary Figure 1

Supplementary Figure 2

Supplementary Table

Supplementary Figure 3
