# Supplementary material for: Drug discovery for male subfertility using high-throughput screening: a new approach to an unsolved problem
Source: Hum Reprod. 2017 Mar 16;32(5):974–84. doi: 10.1093/humrep/dex055 (PMC5850465; doi:10.1093/humrep/dex055)
Supplement: Supplementary Figure 1 [file dex055_supplementaryfigures1.pdf]

Human Reproduction, 2017
doi:10.1093/humrep/dex055

human reproduction

SUPPLEMENTARY DATA

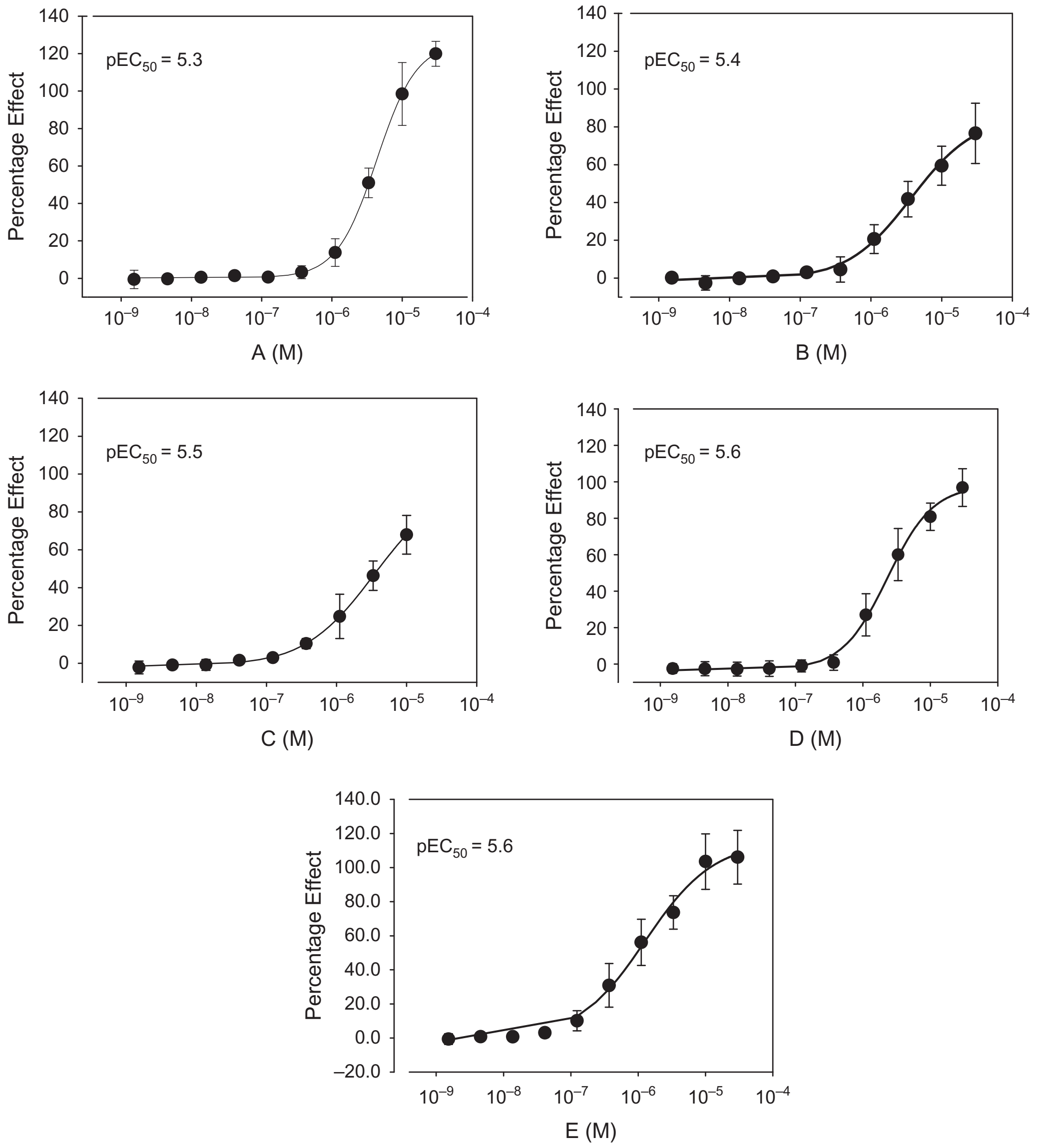

Supplementary Figure S1 Dose effect curves. Best five agonist compounds following ion channel library screening: DDD00104789, DDD00104960, DDD00105020, DDD00105498 and DDD00106181, subsequently coded A–E.
