# Supplementary material for: Drug discovery for male subfertility using high-throughput screening: a new approach to an unsolved problem
Source: Hum Reprod. 2017 Mar 16;32(5):974–84. doi: 10.1093/humrep/dex055 (PMC5850465; doi:10.1093/humrep/dex055)
Supplement: Supplementary Figure 2 [file dex055_supplementaryfigures2.pdf]

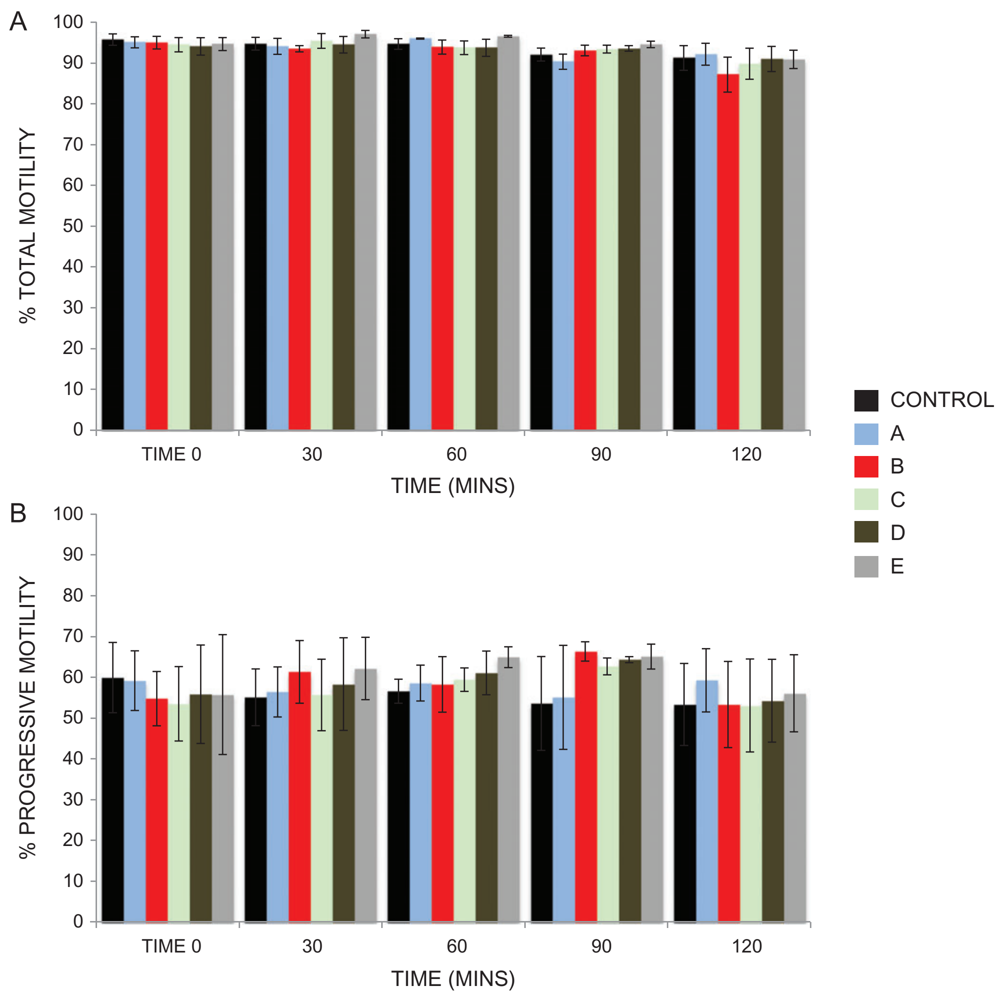

Supplementary Figure S2 CASA kinematic data in sperm following preparation by DGC. No significant difference in % total motility (**A**) or % progressive motility (**B**) seen with 10 µM compound A–E compared with control over time: time 0 (T0) to 120 min. No significant difference in average path velocity (VAP; **C**), curvilinear velocity (VCL; **D**), straight-line velocity (VSL; **E**) or lateral head displacement (ALH; **F**) following incubation with 10 µM compound A over time: time 0–120 min. No significant difference in VAP (**G**), VCL (**H**), VSL (**I**) or ALH (**J**) following incubation with 10 µM compound B over time: time 0–120 min. CASA, computer-assisted sperm assessment; DGC, density gradient centrifugation.

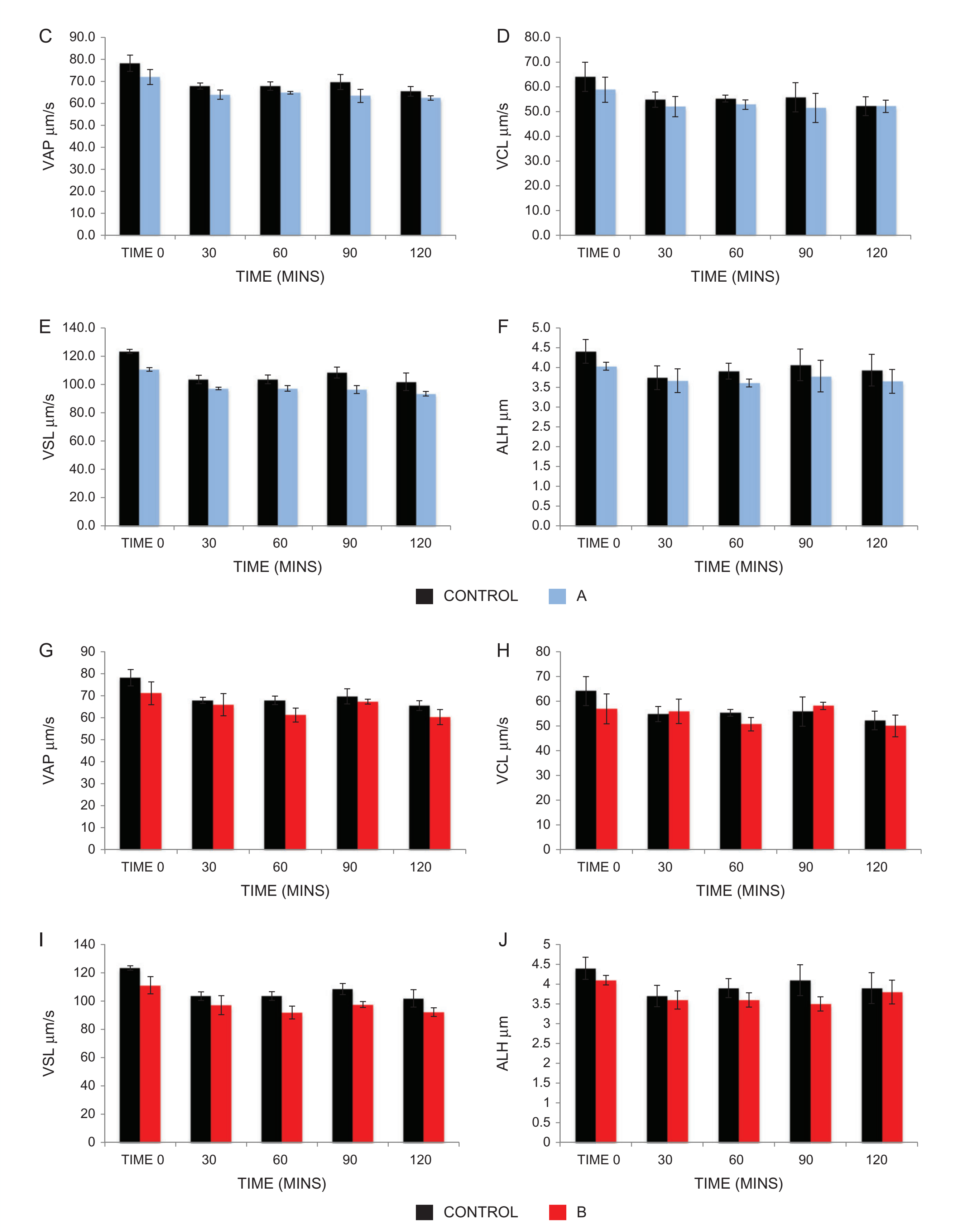

Supplementary Figure S2 *Continued*
