# Supplementary material for: Drug discovery for male subfertility using high-throughput screening: a new approach to an unsolved problem
Source: Hum Reprod. 2017 Mar 16;32(5):974–84. doi: 10.1093/humrep/dex055 (PMC5850465; doi:10.1093/humrep/dex055)
Supplement: Supplementary Table [file dex055_supplementarytablesi.pdf]

Supplementary Table SI Diagnostic semen analysis parameters for patients (n = 17) undertaking fertility treatment (IVF/ICSI).

Research ID	Diagnostic semen analysis				After DGC	
	Volume (ml)	Conc. (M/ml)	Progressive motility (%)	Normal forms (%)	Conc. (M/ml)	Progressive motility (%)
R1333	3.7	38.0	49	3.0	10.3	98.5
R1346	7.0	16.9	72	7.5	6.0	95
R1347	4.7	3.7	32	1.4	0.73	15
R1348	1.0	80.0	55	8.0	29.0	95
R1357	3.2	60.0	59	4.0	16.3	95
R1364	1.5	35.0	74	14.0	7.0	95
R1371	2.4	65.8	56	5.0	3.7	85
R1384	4.8	86.5	28	2.8	5.7	90
R1385	0.9	34.3	44.8	3.7	9.0	95
R2580	1.6	188.0	48.0	8.0	2.9	95
R2581	2.6	15.0	36.0	0.3	2.5	90
R2614	3.8	46.0	54.0	3.0	3.0	93
R2615	1.6	51.0	85.0	9.0	27.1	90
R2622	2.0	65.1	43.0	11.0	19.0	92
R2623	4.0	15.0	64.0	2.0	6.0	95
R2632	2.8	89.0	60.0	3.0	6.85	98
R2634	6.0	31.6	47.0	5.0	1.0	90

Samples surplus to clinical requirements were donated for research on the day of treatment at Ninewells Assisted Conception Unit. Sperm concentration and progressive motility following standard preparation by density gradient centrifugation (DGC) are also shown. All patients underwent IVF, except R1347 (ICSI).
