# Supplementary material for: Drug discovery for male subfertility using high-throughput screening: a new approach to an unsolved problem
Source: Hum Reprod. 2017 Mar 16;32(5):974–84. doi: 10.1093/humrep/dex055 (PMC5850465; doi:10.1093/humrep/dex055)
Supplement: Supplementary Figure 3 [file dex055_supplementaryfigures3.pdf]

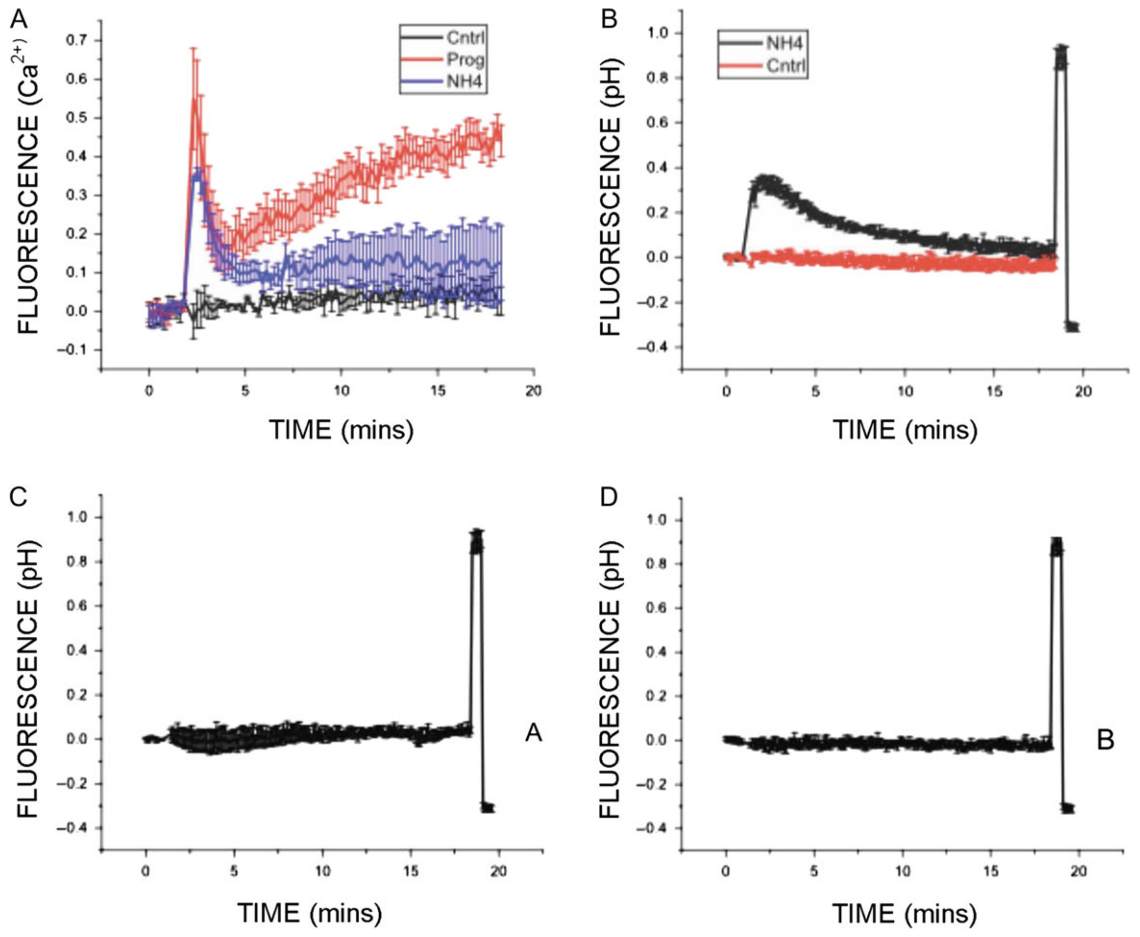

Supplementary Figure S3 FLUOstar data. FURA2 was used to measure intracellular Ca^{2+}. BCECF (2′,7′-bis(2-carboxyethyl)-5,6-carboxy-fluorescein) was used to track changes in intracellular pH. (**A**) Expected calcium response following addition of 3.6 μM progesterone (red), NH_4 (blue) or control (black). (**B**) pH change following addition of NH_4 (black) and control (red). No effect on pH was observed following addition of compound A (**C**) or B (**D**).
